# The Safety of Digital Mental Health Interventions: Findings and Recommendations From a Qualitative Study Exploring Users’ Experiences, Concerns, and Suggestions

**DOI:** 10.2196/62974

**Published:** 2025-02-07

**Authors:** Rayan Taher, Daniel Stahl, Sukhi Shergill, Jenny Yiend

**Affiliations:** 1 Department of Psychosis Studies King’s College London London United Kingdom; 2 Department of Biostatistics and Health Informatics King’s College London London United Kingdom; 3 Kent and Medway Medical School Canterbury United Kingdom

**Keywords:** digital mental health, safety, user perspective, patient perspective, qualitative, risks, risk mitigation, deterioration, nonresponse, data safety

## Abstract

**Background:**

The literature around the safety of digital mental health interventions (DMHIs) is growing. However, the user/patient perspective is still absent from it. Understanding the user/patient perspective can ensure that professionals address issues that are significant to users/patients and help direct future research in the field.

**Objective:**

This qualitative study aims to explore DMHI users’ experiences, views, concerns, and suggestions regarding the safety of DMHIs.

**Methods:**

We included individuals aged 18 years old or older, having experience in using a DMHI, and can speak and understand English without the need for a translator. Fifteen individual interviews were conducted. Deductive thematic analysis was used to analyze the data.

**Results:**

The analysis of the interview transcripts yielded 3 main themes: Nonresponse: A Concern, a Risk, and How Users Mitigate It, Symptom Deterioration and Its Management, and Concerns Around Data Privacy and How to Mitigate Them.

**Conclusions:**

The results of this study led to 7 recommendations on how the safety of DMHIs can be improved: provide “easy access” versions of key information, use “approved by...” badges, anticipate and support deterioration, provide real-time feedback, acknowledge the lack of personalization, responsibly manage access, and provide genuine crisis support. These recommendations arose from users’ experiences and suggestions. If implemented, these recommendations can improve the safety of DMHIs and enhance users’ experience.

## Introduction

Digital mental health interventions (DMHIs) are mental health interventions that are delivered through digital platforms such as mobile apps, websites, or virtual reality [1]. Some of the added benefits of mental health interventions that are delivered digitally are improved accessibility, scalability, convenience, and the potential for anonymous engagement [2]. To realize these benefits, users need to trust that these interventions are effective and safe [2]. The evidence shows that DMHIs can be as effective as traditional face-to-face therapies, especially for common mental health disorders such as depression and anxiety [3,4]. However, the safety of DMHIs is still an evolving field [1,2,5,6].

DMHI’s users face similar risks to those in face-to-face therapy, such as deterioration in symptoms, novel symptoms (experiencing new mental health symptoms during treatment), and nonresponse [1]. Deterioration of symptoms, observed in approximately 3%-10% of psychotherapy cases [7,8], signifies a phenomenon where patients’ conditions worsen during therapy. Deterioration is the most common side effect of mental health therapies (face to face and digital) [1]. There is a debate in the literature about whether deterioration is a normal and integral part of therapy or an unnecessary side effect [9,10]. A recent experts’ consensus study concluded that short-term deterioration that occurs during therapy is part of therapy and should not be considered a safety concern; however, deterioration still needs to be monitored to ensure that it is not chronic, severe, and does not lead to an adverse event such as the patient dropping out of treatment [11]. Nonresponse occurs when the therapy is not effective in relieving the target symptoms [12]^.^ It is considered a negative outcome as it hinders access to more effective treatments, spontaneous remission, and may prolong or even increase distress [12]. Additionally, the digital nature of DMHIs introduces additional risks to mental health therapies such as technical issues and privacy concerns [5,13].

The expanding body of literature addressing the safety of DMHIs is noteworthy [1,5,6]. While considerable attention has been devoted to exploring the safety of DMHIs (how it is assessed, analyzed, and reported), a notable gap exists in the qualitative understanding of individual perspectives on the topic. Existing qualitative studies in this field have either focused on the viewpoints of health professionals and medical students [14,15] or have sought user opinions on specific digital innovations usually as part of a wider program of development work. A few studies have sought service users’ views on digital interventions more generally [16-18], but no work to our knowledge has investigated user/patient perspectives specifically about the safety of these technologies [19,20]. Understanding the user/patient perspective on safety can help direct future research in the field to ensure professionals focus on issues significant to users/patients; identifying users’ concerns can help professionals address these issues, leading to higher rates of adherence and engagement. For that reason, this qualitative study aims to explore DMHIs users’ experiences, views, concerns, and suggestions regarding the safety of DMHIs.

## Methods

### Design and Aim

This qualitative study utilized individual interviews to explore users’ experiences, views, concerns, and suggestions about the safety of DMHIs. The Consolidated Criteria for Reporting Qualitative Research (COREQ) was used to report the results of this study [21].

### Recruitment

The study included individuals aged 18 years or older, with previous experience using a DMHI, and speaking and understanding English without the need for a translator. The DMHI needed to be a mental health intervention that was provided via a tech-based medium (eg, app, website, virtual reality) and targeted a specific mental health condition. Participants were recruited using nonpurposive sampling by posting advertisements on authors’ own professional social media platforms, such as X (formerly Twitter; X Corp.) and LinkedIn (Microsoft Corporation), an online participant recruiting platform (MQ; MQ Mental Health Research), the university’s (King’s College London) research volunteering circular email, and through DMHI trials whose participants consented for their details to be shared for future research. A total of 54 potential participants reached out; 15 (28%) were eligible and participated in the study, 7 (13%) did not complete the online eligibility form, and 32 (59%) were ineligible. Eligibility was determined through screening questions asked of potential participants to gather further details about the specific intervention used. The main reasons for ineligibility were that the intervention used was telehealth (eg, face-to-face therapy conducted via video call) or did not target a specific mental health condition (eg, mindfulness apps).

### Participants

A total of 15 participants were recruited to participate in this study. Researchers initially estimated a sample size of 6-16 participants based on pragmatic recommendations from the literature, suggesting that 6-16 interviews provide sufficient information power [22]. Then the final sample size (15 participants) was determined based on the richness of the data and their ability to sufficiently answer the research question [23]. Of the 15 participants, 12 (80%) were females and 3 (20%) were males. All participants lived in the United Kingdom at the time of the interviews. Participants had an average age of 30 years (SD 6.43 years; range 19-42 years). On average, participants had used a DMHI for 8 months (SD 10.51 months; range 1-36 months). The DMHIs used by participants in this study were Beating the Blues, Calm Harm, FREED-M, Happify, Molehill Mountain, Moodkit, Silver Cloud, Sleepio, STOP app, Woebot, Youper, and an online intervention for Bulimia. See Table 1 for more information on the interventions used by participants in this study. Some participants used the DMHI for both anxiety and depression. Other than that, no other participants used the DMHI for more than 1 target symptom or condition.

**Table 1:** The DMHIs^a^ used by participants in this study (N=15).

DMHI		Values, n (%)	Duration (months) of use, mean (range)
**DMHI’s target symptom/condition**			
	Depression and anxiety	5 (33)	3.4 (1-6)
	Depression	2 (13)	19.25 (2.5-36)
	Self-harm	2 (13)	12.75 (1.5-24)
	Paranoia	1 (7)	3 (N/A^b^)
	Insomnia	1 (7)	1.5 (N/A)
	Postnatal depression	1 (7)	24 (N/A)
	Bulimia	1 (7)	1.5 (N/A)
	Anxiety in autism	1 (7)	3 (N/A)
	Eating disorders	1 (7)	3 (N/A)
**DMHI’s format**			
	Web-based	7 (47)	2.5 (1-6)
	App-based	6 (40)	11.5 (1.5-36)
	Artificial intelligence chatbot	2 (13)	15 (6-24)
**Therapist involvement**			
	Self-administered (users independently used the DMHI without any support)	12 (80)	9.33 (1.5-36)
	Hybrid (users independently used the DMHI while receiving regular support)	3 (20)	1.66 (1-2)
**How participants found the DMHI**			
	Health care professional	7 (47)	N/A
	Social media	2 (13)	N/A
	App store	2 (13)	N/A
	DMHI research	2 (13)	N/A
	Work (via human resources)	1 (7)	N/A
	University website	1 (7)	N/A

^a^DMHI: digital mental health intervention.

^b^N/A: not applicable.

### Materials

Individual interviews with participants were conducted and recorded online via Microsoft Teams (Microsoft Corporation). Interviews were semistructured. See Multimedia Appendix 1 for the topic guide. The interviewer (RT) used prompts to facilitate and guide the discussion. RT has experience conducting individual interviews for research purposes.

### Procedure

Participants viewing the study advertisement were asked to email the researcher if they were interested. The researcher replied to introduce them to the study, share the participant information sheet (PIS), and request that they complete an online (Qualtrics) questionnaire to check eligibility. The same researcher contacted eligible participants to check that they had read the PIS, answered any questions that they had, and asked if they were interested in participating in the study. Those expressing a desire to participate were emailed an online form that includes a few questions (details below) and an e-consent form (using Qualtrics) to sign and a link to book a 1-hour slot for the interview. The online form asked for demographic details such as gender and age, details about the intervention (name and intended purpose), and how long they used the intervention for. All interviews were audio recorded. At the end of the interview, participants were sent a thank you email and a £20 (US $25) voucher as compensation for their time. Interviews lasted on average 40 minutes. Recordings were automatically transcribed by Microsoft Teams and were verified for accuracy by RT. Once transcription was complete all recordings were deleted.

### Analysis

#### Thematic Analysis Process

The 15 transcripts were uploaded onto NVivo (Lumivero, LLC) for analysis [24]. We used deductive thematic analysis to analyze the data. Thematic analysis is a process used to identify patterns or themes within qualitative data to answer or explore a research question [25,26]. The analysis was conducted collaboratively by 2 researchers (RT and JY), who followed Braun and Clarke’s [25] step-by-step guide to conduct a thematic analysis by familiarizing themselves with the 15 transcripts and coding the data. They then organized the codes based on relatedness, reviewing them, and defining and naming them as subthemes and themes [25]. The thematic analysis acknowledges “the researcher’s reflective and thoughtful engagement with their data, and their reflexive and thoughtful engagement with the analytic process is essential” [25]. It recognizes the potential benefits of using multiple coders, such as achieving richer interpretations, however, it does not view this as a requirement [26]. Researchers are discouraged from attempting to provide accounts of “accurate” or “reliable” coding or pursuing consensus among multiple coders [26].

Given the research question and acknowledging that participants’ experiences and perspectives on safety may vary in this study, the analysis was used to reflect the range of experiences of participants and highlight how these might differ, rather than attempting to merge these experiences into a single, unified interpretation [25]. Once the results of the study were ready, they were shared with all 15 participants to review and ensure that they were representative of their experiences. Three participants responded and said that they agreed with the results and did not offer any additional insights or suggest any alterations.

#### Researcher Reflexivity

A critical realist epistemology was adopted for this study, where the researchers aimed to explore participants’ subjective experiences, acknowledge them as “real,” and recognize the researchers’ inability to fully access that reality [25]. The researchers were aware of their reflexivity [27]; at the time of this study, they all worked on a separate clinical trial that aimed to assess the efficacy and safety of a specific DMHI. The first author (RT) was completing her PhD on the safety of DMHIs. This study was 1 of 4 separate pieces of work for the PhD (other work comprising a systematic review, a methodology paper, and an experts’ consensus study). This study was not directly related to or in any way part of the clinical trial that researchers were working on. As a team, the researchers were invested in learning how users/patients experience risks, react to them, and what risks matter to them in order to contribute to the field and improve their approach to safety.

### Ethical Approval

Ethical Clearance was provided for this study by the King’s College London (reference number LRS/DP-22/23-35403).

## Results

### Overview

The analysis of the data using deductive thematic analysis led to 3 major themes:

- Nonresponse: A Concern, a Risk, and How Users Mitigate It
- Symptom Deterioration and Its Management
- Concerns Around Data Privacy and How to Mitigate Them

### Theme 1: Nonresponse: A Concern, a Risk, and How Users Mitigate It

#### Assessing the Effectiveness of DMHIs

Under this theme, participants spoke about their concerns regarding the DMHI being ineffective, experiencing ineffectiveness/nonresponse as a risk, and the methods they used to assess whether a DMHI was safe and effective.

#### Concerns Around Nonresponse

Users of DMHIs were concerned about the potential ineffectiveness of these interventions. Will these interventions be able to help them? Are these interventions evidence-based? One important area of concern is illustrated as follows:

Umm, I was concerned with like how helpful it would actually be, being that it is an online thing and like I'm not actually talking to a person you know...I was even actually concerned when I started the first session, whether the program would have lasting effects on actually helping me or supporting my mental health.

Participant 6, used a DMHI for depression for 2.5 months

It is likely that participants were doubtful about their interventions’ effectiveness because they were struggling and in such emotional pain that they were unsure how a technology with no human could alleviate their pain and improve their mood.

#### Nonresponse as a Risk

Participants also spoke about the risk of the DMHI being unhelpful and ineffective, and how that at times led to further frustration, deterioration, and self-blame, for example:

I found it ineffective, if I'm honest. I think it was the set of six or eight weeks...I remember that the second week I burst into tears. It just felt so pointless. I can't remember what set me off, but it just felt so pointless.

Participant 9 used a DMHI for an eating disorder for 1.5 months

Another participant explained how the ineffectiveness of the DMHI led to a deterioration in her symptoms and feelings of isolation and self-blame. She said:

So, it's sort of added to that frustration when it was making the situation worse...So now, I felt like it would lead me to do sort of negative coping strategies...I'd be like get angry and irritable with people, or I'd go and overeat....it definitely sort of furthered the thoughts that there was like no one to help me...It made low moments even worse...It almost triggered sort of thoughts of like, oh, something's wrong with me. Why can't the program help me?

Participant 14 used a DMHI for post-natal depression for 24 months

#### Users’ Method for Assessing Safety and Effectiveness

Users used 2 main methods to assess whether they thought a DMHI was safe and effective: (1) social proofing, which refers to the tendency to follow the behavior of others as a guide for one’s own actions [28]; and (2) assessing the contribution of experts or a trusted body. Some participants opened up about finding it difficult to assess the safety of a DMHI, and not knowing how to do that.

Participants wanted to know that professionals were involved in the development of the DMHI and that scientific research has been conducted to assess it, saying:

I suppose I would want to know how it was developed and in partnership with mental health practitioners...so I think with credibility, I guess things like whether they have worked with the university or with kind of recognized academics and done any kind of scientific research and rather than just user testing.

Participant 11 used a DMHI for insomnia for 1.5 months

In this participant’s case in particular, knowing that the intervention was evidence-based was very important because she had struggled with insomnia for more than a decade and had tried many things (face-to-face therapy and medication) that were not helpful for her. Users also relied on other users’ experiences. They checked reviews, ratings, and social media groups to find out more about the DMHI. The following quote is an example of this:

Uh, I have this habit of looking up these things online, so I would look up reviews of the app online. Maybe even check out any details about how helpful it has been. If people have had good experiences, bad experiences, they've had negative experiences, what have they been about and how I could avoid them. I would maybe even like look at Facebook groups.

Participant 6 used a DMHI for depression for 2.5 months

Some participants were honest about not knowing how to check the safety of the DMHI, and needing the support of professionals to be able to do so, they said:

I mean, unless it was like referred by my GP or kind of, you know, promoted through kind of official channels like the NHS website or something, I don't know how I would even check that an app has all the right checks, and you know safeguarding approvals or whatever.

Participant 13 used a DMHI for depression and anxiety for 2 months

Other participants suggested that the DMHI needs to assess users’ suitability, saying:

I guess before someone's able to access the app kind of going through, I don't know, some sort of risk assessment on like who would find it useful.

Participant 7 used a DMHI for paranoia for 3 months

Another participant thought that this could be achieved by the DMHI clearly stating its intended use and target population:

I think the app would need to be really explicit about the limitations and sort of say up front like this is not for severe mental health issues or this is for maintenance.

Participant 14 used a DMHI for post-natal depression for 24 months

### Theme 2: Symptom Deterioration and Its Management

#### Addressing Symptom Deterioration in DMHIs

Under this theme, participants spoke about experiencing symptom deterioration. Participants also gave their feedback on one of the methods used to support them when experiencing or struggling to cope (referral to other services and crisis support) and their suggestions on how deterioration can be managed.

#### Deterioration of Symptoms

Participants talked about how using a DMHI and dealing with their mental health struggles at times led to a deterioration in their mental health symptoms because it made them think about things that were upsetting. One participant who was struggling with sleep said:

I did find thinking more closely about my trouble with sleep did initially make me more anxious about sleep and made it harder to sleep. So, like the kind of tracking and then realizing that actually that was a really bad night...sometimes makes it harder to sleep the next night, by bringing it to the forefront.

Participant 11 used a DMHI for insomnia for 1.5 months

It is important to note that this participant found her intervention effective in helping her manage her insomnia and improve her sleep quality. This aligns with recent findings from an expert consensus study, which concluded that symptom deterioration is not a safety concern of DMHIs but rather a normal part of therapy [11].

In some cases, the inflexibility of the predetermined content in the DMHI and its inability to cater specifically to each user’s needs (ie, lack of personalization) meant that the DMHI was unable to relate to users’ emotional state and could lead to a deterioration in symptoms, for example:

So sometimes the AI (chatbot) like would give me suggestions that didn't really fit my situation. So, I'm like, you know what? Forget it. I'm not even going to do it, and I would feel worse afterwards because I wanted to express it. And then I'm just sitting here typing things, and it's not helping. It wasn't really built to recognize that CBT isn't effective for certain situations.

Participant 14 used a DMHI for post-natal depression for 24 months

#### Managing Deterioration

As deterioration is the most common negative effect of DMHIs [1], the authors asked participants how they think DMHIs could support them through it. Some participants said that normalizing deterioration would be very helpful. Participant 1 explained how that could be done in a hybrid model:

I think one way to support...is to have a video call therapy session. With an agent for example to make me understand that these things are normal. So, at that I would be reassured that I'm getting back to normal.

Participant 1 used a DMHI for depression for 36 months

Another participant explained how that could be done in a nonhybrid model:

Uh, maybe you know if the app had a mood tracker. The algorithm could check if you’re feeling low right after therapy. It might just send them a message “Hey, if you're feeling down, you just check, you just had therapy. This could be normal” and that would be like, uh, fair enough.

Participant 5 used a DMHI for depression and anxiety for 1 month

Another participant suggested using regular check-ins to detect deterioration and provide users with support accordingly, saying:

Umm, I think the biggest thing is regular check-ins. That's quite an important thing about how they're finding it and what particularly is so difficult, maybe even not slowing it down, but having a bit of flexibility around kind of OK, you found this section of the app pretty difficult.

Participant 10 used a DMHI for self-harm for 1.5 months

#### Signposting to Other Services for Support

Most participants (11/15, 73%) were provided with emergency numbers, within the DMHI, to call in case they felt that they could not cope and needed further support. Some participants shared that they found this support helpful:

They gave me the contact numbers of like mind and Samaritans in case I needed urgent help. I'm using an online service. If I did need help, I could contact these services, which is actually really helpful because once or twice when I really felt like I was troubled at night, this did come in handy.

Participant 6 used a DMHI for depression for 2.5 months

However, other participants felt that the signposting to crisis support within the DMHI was tokenistic, sharing:

It's hard to feel like it's a genuine thing. It feels almost like a boilerplate that they put in every conversation. It doesn't really feel like there's thought going into it like “Ohh I recognize that the program can't help you with this. This would be better for a psychiatrist.

Participant 14 used a DMHI for post-natal depression for 24 months

Further analysis of these data highlighted that the participants who had a positive experience with the DMHI (ie, found it helpful) experienced the referral to crisis support information positively. By contrast, participants who found the DMHI unhelpful and were frustrated with it found the crisis support information unhelpful and ingenuine. Thus, users’ relationship with the DMHI and their feelings toward it informed how they felt about being referred to other services. Users who found the DMHI helpful were likely to view the information about other services as a helpful bonus, whereas those who found their DMHI unhelpful were likely to doubt its genuine concern for them and thus viewed such referrals as a mere box-ticking exercise.

### Theme 3: Concerns Around Data Privacy and How to Mitigate Them

#### Participant Perspectives on Data Privacy

Under this theme, participants spoke about their concerns around data privacy and their suggestions on how to help ease these concerns.

#### Concerns Around Confidentiality and Data Privacy

Participants were concerned about their data. Was the DMHI confidential? If not, who is it sharing their data with? Participants talked about their concern that the data might be shared with their health care team without their consent:

I had concerns about the information that I was putting in, and the kind of data that might have been collected. It's kind of that worry that what you're writing isn't actually confidential or you know that it could go back to someone else. I think one of the biggest worries for me was that what I was inputting in the app might have been given to my psychologist or somehow, you know, connected with the NHS or something like that.

Participant 10 used a DMHI for self-harm for 1.5 months

It is worth noting that, given the sensitivity of participant 10’s struggle with self-harm, it is understandable why they were particularly concerned about their data being shared—even with their health care provider.

#### A User-Friendly Data Policy

Participants expressed their frustration with the vagueness and complexity of how DMHIs present their data protection policy and had suggestions on how that can be improved. Some thought that DMHIs should make key data protection information available in a simpler and more readily accessible format:

Maybe just share more information...like make it clear what you do to protect users' data. I don't want to have to go through, you know, all of your Terms and conditions, privacy policies and things like that to find out what it is. I mean, nobody's actually going to do that. I would never actually do it, so it would be helpful if it was just clearly mentioned somewhere.

Participant 6 used a DMHI for depression for 2.5 months

Another participant said that all they wanted from a DMHI is to be honest about what data they are storing and why:

They could kind of like emphasize maybe that your data isn't stored under an identifiable name that leads to you. Or say we are storing the data, but it is confidential and we're doing it to help more people. That's all...just being open about it.

Participant 8 used a DMHI for an eating disorder for 3 months

It is worth noting that the data from this study did not show any association between duration of use and participants’ experiences. Participants had mixed responses to the DMHI when they used it for a short period (1.5 months, eg, participants 10 and 11), and those who used the DMHI for a long period (24 months; eg, participant 14) did not necessarily have a good experience. However, Table 1 does show that the DMHIs that were targeting low mood–related areas were used for the longest period, such as depression (on average 19.25 months), self-harm (on average 12.75 months), and postnatal depression (on average 24 months). It is also noted that self-administered interventions were used for longer periods compared with hybrid interventions (on average 9.33 months vs 1.66 months). This is expected given that self-administered interventions require fewer resources and clinician time.

## Discussion

### Advancing Knowledge on DMHI Safety

This research was successful in exploring and understanding users’ experiences, views, concerns, and suggestions regarding the safety of DMHIs. Until now, such findings have been absent from the literature. These findings contribute to advancing the field of digital mental health safety by providing valuable evidence of the viewpoints and experiences of its target population.

### Principal Findings

#### Overview

**Figure 1:**
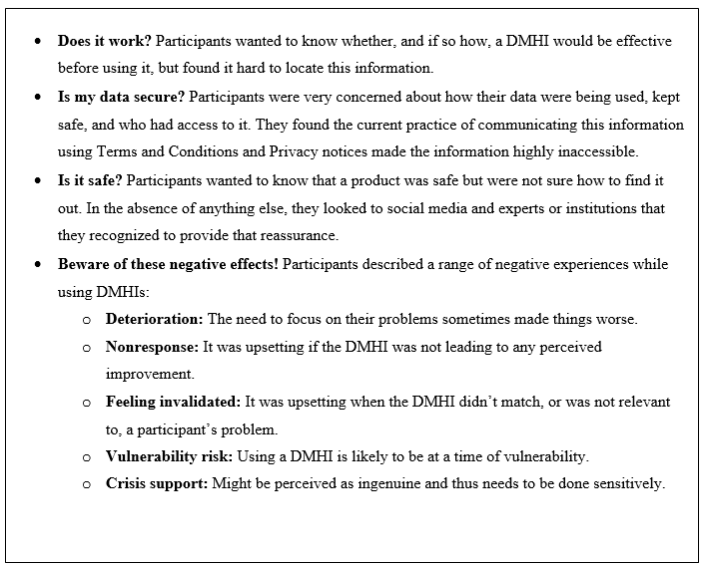
Key findings (in lay language). DMHI: digital mental health intervention.

The main findings of this study are presented in Table 2 (also see Figure 1) using user-friendly language and in the form of recommendations.

**Table 2:** User-informed recommendations to improve DMHI^a^ safety.

Recommendations	Description
1. Provide ‘easy access’ versions of key information	Ensure that DMHI product manufacture and approval include a requirement to provide readily accessible, easy-to-read lay summaries of key information. At a minimum, these should cover (1) evidence of effectiveness; (2) data usage, security measures, and access (ie, who can access the data); and (3) potential negative effects.
2. Use ‘Approved by...’ badges	Introduce a sectorwide, widely recognized, branded badge to provide top-level reassurance of the quality and safety of any DMHIs bearing that badge.
3. Acknowledge the lack of personalization	DMHIs should flag to users their inability to be fully personalized and adaptable to an individual user’s needs to mitigate feelings of invalidation and disappointment.
4. Anticipate and support deterioration	Before using a product, users should be alerted to possible mood or symptom deterioration, given normalizing information, and signposted to relevant support to help mitigate these effects should they occur.
5. Provide real-time feedback	DMHIs should internally track users’ progress and provide feedback on whether they are benefiting as expected. Where there is no individual benefit, despite the appropriate use of the product, users should be automatically advised to seek alternative support.
6. Provide genuine crisis support	Content should include an acknowledgment of the DMHIs’ limitations and a summary of each crisis service’s support. It is recommended to consult the target population on wording to ensure genuine concern for users is communicated.
7. Responsibly manage access	DMHIs should incorporate an assessment of suitability focusing on risk levels and the appropriateness of the intervention for users’ specific mental health conditions and severity. This could be done with a simple set of initial built-in questions which output a recommendation to use, or not use, the product based on the user’s response.

^a^DMHI: digital mental health intervention.

#### Users’ Concerns Around the Safety of DMHIs

In this study, users expressed 2 primary concerns regarding the safety of DMHIs: (1) whether a product would be effective and (2) whether their data would be secure and confidential. Evidence around the effectiveness of DMHIs is usually disseminated in academic peer-reviewed articles. This poses an accessibility challenge for the typical users, which is only slightly tempered by recent initiatives toward making open-access publications the norm in academia. It has been documented that uncertainty around the effectiveness of a DMHI is a key barrier to its use [15]. To address users’ concerns about effectiveness, it is crucial to translate scientific findings into lay language and publish them in user-friendly formats to improve accessibility (recommendation 1). This dovetails well with the increasing requirements placed upon academics to evidence the impact of their work.

Regarding users’ second major concern, data safety and confidentiality, participants in this study suggested that DMHIs provide their users with clear and concise information on how their data are used, stored, and who has access to it. Recognizing that users often overlook traditional lengthy privacy policies and terms and conditions [29], DMHIs could supplement these by providing users with a layperson’s summary of how their data are being used, kept safe, and who has access to them (recommendation 1). Clear and transparent communication about data and privacy would help build trust, a key component of the therapeutic relationship [30]. The evidence to date suggests that the digital therapeutic alliance is both relevant and important in DMHIs [19,20].

#### Assessing a DMHI’s Safety From Users’ Perspective

It is important to understand how users assess the safety of DMHIs, as this will inform professionals about where users look for safety information and so where and how best to provide it. Participants in this study described methods for assessing the safety of a DMHI that reflected social proof, a concept first attributed to Robert Cialdini [31,32]. Social proof refers to situations where people use opinions and information from others similar to themselves to influence personal choices, decisions, and behaviors, especially if uncertain [31,32]. In the present context, this involved reading online product reviews on websites and mobile app stores. Other participants expressed that they did not know how to assess the safety of a DMHI. In a different qualitative study, medical students shared a similar experience, expressing difficulty in identifying which DMHIs were evidence-based [15]. These students also criticized the lack of guidance available for users on how to find evidence-based DMHIs [15].

There are already regulatory bodies equipped and responsible for assessing the efficacy and safety of DMHIs and making recommendations for use. In the United Kingdom, this includes NICE (The National Institute for Health and Care Excellence) and the MHRA (Medicines and Healthcare Products Regulatory Agency). The NHS (National Health Service) previously had a health app store called the “NHS Health Apps Library,” but it was decommissioned in 2021 due to the increasing complexity of maintaining the library and ensuring the safety and effectiveness of the listed apps. An alternative to this has emerged through the Health App Library provided by the Organization for the Review of Care and Health Apps (ORCHA), in collaboration with health providers such as NHS Trusts. This library offers a list of mobile apps that have been reviewed by ORCHA for effectiveness and safety. One effective way to demarcate a product’s safety and efficacy status would be for regulators to introduce a branded, recognizable stamp or badge to be displayed by products achieving prespecified minimum safety and efficacy requirements (recommendation 2). What those requirements should be, however, would entail significant additional research to achieve a consensus across industry, regulatory bodies, academia, developers, and users. Nevertheless, this approach would capitalize on users’ existing tendency to seek safety information via the product’s mobile app page or website and is therefore likely to ultimately be an effective means of disseminating key information on which end users can base their decisions. A notable advancement in this direction is Google’s revision of their app store’s health policy, which mandates that starting from May 31, 2024, all health apps posted on their app store must prove compliance with relevant laws and regulations (privacy policy, ethics approval, and certification when required) [33].

#### Risks Experienced by DMHIs’ Users and Their Suggestions on How to Mitigate Them

Participants in this study spoke about 3 risks they had experienced as a result of using a DMHI: feeling invalidated by the DMHI, deterioration in their symptoms, and nonresponse. Users noted that, unlike a human therapist, the DMHIs’ inability to be fully responsive, personalized, and adaptable to each user’s needs left them feeling invalidated and unheard. Such experiences undermine the therapeutic relationship. There is evidence that personalization in a DMHI fosters therapeutic alliance [20], and thus the lack of it is likely to undermine this alliance. The weaker the digital therapeutic relationship, the more this is likely to undermine the effectiveness of the DMHI [30]. In a qualitative study involving Australian psychologists and their experiences with DMHIs, the psychologists expressed the view that DMHIs are inferior to face-to-face therapy due to their limited capacity for personalization [14]. In another qualitative study, medical students made the same comparison between DMHIs’ and health professionals’ ability to provide personalized therapy [15]. This is a limitation of current technologies, which might change with the future advances of artificial intelligence. However, for now, it is important for DMHIs to acknowledge and communicate this limitation to users to mitigate feelings of invalidation (recommendation 3).

Participants suggested that DMHIs can support users experiencing deterioration by informing them about the possibility, normalizing it, and providing pathways to relevant support when it occurs (recommendation 4). These suggestions align with those made by digital mental health professionals in a recent consensus statement [11]. Other studies have suggested implementing an automated process within DMHIs to monitor and flag when participants’ symptoms deteriorate beyond a predefined threshold [5]. Although that threshold would be subject to individual conditions and clinical opinion within any specific context, a useful starting point would be to adopt the clinical “rule of thumb” that considers a 20% change in symptoms as a meaningful variation [34].

“Nonresponse” is a documented potential side effect of DMHIs [1,12]. It was interesting to see how nonresponse from users’ perspective was almost a compound negative effect that led to a cascade of unwanted effects including feelings of frustration, hopelessness, deterioration, isolation, and self-blame. One way to address this would be for DMHIs to track users’ clinical outcomes, identify those experiencing nonresponse, and provide them with targeted pathways to further support as a way of mitigating these possible adverse consequences (recommendation 5).

#### The Risk Mitigation Methods Experienced by DMHIs’ Users

When discussing how DMHIs mitigate risks and safeguard users, users highlighted the importance of signposting to other sources of support. The majority of users (11/15) were provided with details of emergency numbers and other mental health services; however, not all users found these helpful. Some users felt that this was a checkbox exercise that DMHIs needed to complete and that the crisis support provided was ingenuine. Users of DMHIs may feel this way because of their experiences with mental health services, and because most mental health services/interventions tend to provide crisis support information. Signposting to a different service needs to be done delicately to ensure that the user feels cared for. For that, DMHIs need to be careful about how they present crisis support details. Including an acknowledgment of the DMHI’s limitations, a concern for the user, and a description of the support that each crisis service provides might help make users feel that the intention to provide support is more genuine (recommendation 6). Soliciting user input on how such information is phrased and presented would also be of benefit (recommendation 6).

Only 1 user among our sample of 15 was informed of the potential side effects of the DMHI that they were using. A recent systematic review on the safety of DMHIs found that only one-third of the interventions informed their users of their adverse events or possible side effects [1]. The even lower level of side effect awareness in our study might be due to the commonplace practice of embedding side effect information within inaccessible or often unread documentation (eg, terms and conditions or instructions for use) [6]. Indeed, as reported above, our participants told us that they found these documents particularly impenetrable, suggesting some may have missed out on important side effect information. To ensure user safety, it is important to improve the visibility and accessibility of side effect information by adopting new methods of communication (recommendation 1). This might include a digital equivalent of listing possible side effects on medication labels. Existing regulations already require a digital product label to be displayed within the product itself and this label includes “Cautions” and “Warnings.” It would be a relatively simple matter to add a section “Possible Side Effects” as a further requirement.

However, simply adding information is unlikely, on its own, to meet users’ needs as identified by our study. In addition (and as already discussed earlier), serious consideration should be given to adopting the practice of requiring “easy read” or “lay summary” versions of key information which is provided alongside full and formal versions. This is now standard practice in domains such as academia, governmental, and other public sector organizations. Our study suggests that, as a minimum, this should apply to information on data security and side effects.

Finally, to minimize this risk, our sample of participants/users recommended that DMHIs should evaluate each user’s suitability, with a focus on assessing risk levels and determining the appropriateness of the intervention for their specific mental health condition and severity. These assessments can be included as a standard procedure before onboarding a user onto a DMHI, similar to how patients are screened before they receive face-to-face therapy (recommendation 7).

### Limitations

There are a few limitations to this study. The participants in this study were mostly female (12/15, 80%). The DMHIs used by participants were mostly self-administered (12/15, 80%), and thus results might be biased by their experiences. Additionally, recruiting for a study to explore users’ perspectives on the safety of DMHIs might have attracted individuals who have experienced such issues. It is important to be aware of how the sample of participants in this study could have shaped the results. This is expected in qualitative studies, which aim to explore and understand the experiences and opinions of a sample of the population [35].

### Conclusions

The results of this study led to 7 user-informed recommendations on how the safety of DMHIs can be improved. These recommendations arose from users’ experiences and suggestions. The key findings (Figure 1) and recommendations of this paper could improve the safety of DMHIs, enhance users’ experience, address some of their concerns, and foster a more trusting therapeutic relationship between the user and the DMHI.

## Appendix

Multimedia Appendix 1 Topic guide.

## Abbreviations

COREQ: Consolidated Criteria for Reporting Qualitative Research
DMHI: digital mental health intervention
MHRA: Medicines and Healthcare Products Regulatory Agency
NHS: National Health Service
NICE: The National Institute for Health and Care Excellence
ORCHA: Organization for the Review of Care and Health Apps
PIS: participant information sheet
