# Supplementary material for: The Safety of Digital Mental Health Interventions: Findings and Recommendations From a Qualitative Study Exploring Users’ Experiences, Concerns, and Suggestions
Source: JMIR Hum Factors. 2025 Feb 7;12:e62974. doi: 10.2196/62974 (PMC11845877; doi:10.2196/62974)
Supplement: Multimedia Appendix 1 [file humanfactors_v12i1e62974_app1.docx]

## Supplementary material A

### Topic Guide

#### *Part 1: User’s Experience:*

1. Tell me a bit about the digital mental health intervention(s) that you’ve used?
   - How long did you use it for?
   - Was there a human/therapist support included?
   - Why did you use it?
   - Was it helpful?
   - Can you tell me a bit more about how you heard about it? Did you have any concerns before you downloaded it? (safety)
   - Were you aware of any side effects that the product could lead to?
2. Did you experience any negative effects as a result of using the product? If yes, how did you deal with it?
   - Follow up: Did you know what to do or who to contact?
3. Were you actively asked by anyone whether you experienced a side effect as a result of using the product? How did they do that?
4. Was there anything in the product that you could use if you were struggling or experiencing a side effect?
   - Follow up:
     - Did you receive any instructions on how to use the intervention?
     - Did you know who to contact if you experienced a side effect?
     - Was there an emergency number provided if you needed it?
     - Were you informed about any possible risks? Were there any warnings or safety information?
5. Did you experience any distress or discomfort while you were using the product, even if it was short and passing? How about any deterioration in your mental health?

#### *Part 2: User’s Knowledge:*

1. Let’s say you decided to use a new digital mental health intervention, what would you do to see if it’s safe? What concerns would you have?
2. What do you think are the possible risks of digital mental health interventions? Follow up: Which of these risks concern you the most? What could be done to manage these risk(s) and reassure you?
3. Have you heard of anyone experiencing a side effect after using a digital mental health intervention?
4. Research shows that a common risk of mental health therapies whether face to face or digital is deterioration, meaning that people sometimes feel worse before they feel better. What are your thoughts on this? Do you have a similar experience?
   - Follow up (how can this be mitigated): Hypothetically, let’s say we were going to ask you to use a digital mental health intervention that we know is effective, but we also know that it could lead to “deterioration” I.e., you could feel worse before you feel better. How would you like us to support you? What information would you find helpful?
